# Immunometabolism as predictor of frailty

**DOI:** 10.18632/aging.203737

**Published:** 2021-11-30

**Authors:** Johanna M. Gostner, Blanca Laffon, Thomas K. Felder

**Affiliations:** 1 Institute of Medical Biochemistry, Biocenter, Medical University of Innsbruck, Innsbruck, Austria; 2 Universidade da Coruña, Grupo DICOMOSA, Centro de Investigaciones Científicas Avanzadas (CICA), Departamento de Psicología, Facultad de Ciencias de la Educación, Campus Elviña s/n, A Coruña 15071, Spain; 3 Instituto de Investigación Biomédica de A Coruña (INIBIC), AE CICA-INIBIC. Oza, A Coruña 15071, Spain; 4 Department of Laboratory Medicine, Paracelsus Medical University, Salzburg, Austria

**Keywords:** inflammaging, immunometabolism, frailty, tryptophan, indoleamine-2,3-dioxygenase

Frailty syndrome is a stronger indicator for biological ageing and survival than chronological age. Symptoms include loss of reserves (energy, physical ability, cognition, health), and increased vulnerability to endogenous or exogenous stressors, culminating in a higher risk of negative health outcomes. Less efficient repair mechanisms, insufficient removal of abnormal and degenerated cells, reduced detoxification and deficient immune responses trigger the accumulation of insults. This finally drives “inflammaging”, a state of chronic low-grade immune activation manifested in ageing adults. Inflammaging is a major driving force of frailty, as it contributes to the development of age-related, chronic and degenerative diseases, increased susceptibility to infections and diminished response to vaccines. Therefore, it is of utmost relevance to define biomarkers that may indicate the onset and progression of such processes at early stages. A major challenge is to distinguish accelerated or frailty-related immune activation from immunosenescence processes that intrinsically accompany healthy aging.

Defining abundance, activity and/or differentiation of lymphocyte populations may uncover underlying immunological conditions. Ageing-associated lymphocyte changes include decreased production of T and B cells, diminished export of naive cells to the periphery and relative increases of various memory T cells [1]. Reduced total blood lymphocyte levels or percentages of several subsets were associated with frailty and its severity in different populations of older adults. A cross-sectional study of Spanish older adults reported a significant increase in the CD4^+^/CD8^+^ ratio and a decrease in the percentage of CD19^+^ cells in frail individuals compared to non-frail controls, however only inflammatory plasma markers such as IL-6 and sTNF-RII but not lymphocyte populations progressively increased with frailty severity [2]. Moreover, reduced cytokine production is associated with aging, often correlating with poor prognosis in case of bacterial infection and sepsis.

Metabolites are important systemic mediators of immune homeostasis. They shape the interaction of immune cells with the microenvironment as well as a variety of other processes in parallel. One key example is the accelerated immune-mediated catabolism of the essential amino acid tryptophan along the kynurenine axis by indoleamine 2,3-dioxygenase 1 (IDO-1). The kynurenine to tryptophan ratio in plasma acts as a proxy for IDO-1 activity, if concentrations of inflammatory markers such as neopterin or pro-inflammatory cytokines rise simultaneously. Depletion of tryptophan reduces the proliferation of invading pathogens and under chronic conditions of immune cells, as tryptophan is also the least abundant amino acid in proteins. Persistent reduction of tryptophan is a tolerogenic immunosuppressive mechanism. Kynurenine downstream products may impinge on immune processes, e.g. by selectively inducing apoptosis in lymphocytes. Some are ligands of the environment-sensing aryl hydrocarbon receptor, which is also involved in coordinating activation, differentiation and proliferation processes of immune cells, and hematopoiesis. Moreover, several kynurenine catabolites are neuroactive. Tryptophan is a precursor of the neurotransmitter serotonin, and thus feeds the melatonin pool as well. There is also a regulatory role of tryptophan on protein synthesis in neurodegenerative protein-aggregation. Disturbed tryptophan metabolism is associated with a variety of consequences depending on other underlying conditions, ranging from neuropsychiatric and cognitive disturbances to tumor-associated cachexia and anemia. In addition, the impact of microbial derived-tryptophan and derivatives on the gut-brain-immune axis is of relevance. Furthermore, kynurenine affects skeletal muscle functioning which links tryptophan metabolism to age-related physical impairments.

Certainly, immune-mediated accelerated tryptophan metabolism is part of a larger crosstalk and consequences that are not isolated from other metabolic and redox changes that occur during inflammation. The cytokine interferon (IFN)-γ plays a major role, and some associated pathways are highly susceptible to modulation by lifestyle factors [3, 4].

Human genetic data and various animal models suggest that balanced metabolism of tryptophan may support longer and/or disease-free survival. Several factors along the IDO-1 activation cascade may participate, including polymorphisms of the IFN-γ gene. Increased kynurenine to tryptophan ratios and levels of neopterin, an associated IFN-γ induced marker of cellular immune activation, were predictors of coronary events in the Hordaland prospective health study. Low serum tryptophan in coronary artery disease patients was associated with reduced life expectancy in the Ludwigshafen Risk and Cardiovascular Health study.

Increased tryptophan breakdown and kynurenine levels were observed in frail older adults [5, 6]. These data, and our recent study reporting the association with oxidative DNA damage and a trend to decreased concentrations of vitamin E, highlight the importance of amino acid metabolism and underline that frailty development is linked to a degree of immune activation and oxidative stress beyond what could be expected from biological age alone. Hence, tryptophan and/or its degradation products seem to be suitable candidates for frailty biomarkers [7].

A decrease of serum/plasma total tryptophan and increases of both the kynurenine to tryptophan ratio (and neopterin) were observed also during healthy aging [3, 8]; however sex-related differences and the nutritional status of study participant also play a role. The ratio of free to total tryptophan may be relevant for transport and further metabolic routes, too [3]. A larger systematic analysis of the parameters across lifespan would be needed to gain further information of age-grouped normal concentration ranges in the healthy population.

Altogether, monitoring of tryptophan metabolism and related immunobiochemical pathways provides information on IFN-γ dependent immune activation and can support the early detection of dysregulated immunologic, neuropsychiatric and physiological processes. Robust and cost-efficient analytical methods for the determination of the most important metabolites are available. This may promote personalized strategies to predict and counteract inflammaging, in an early stage and to accompany and to evaluate treatment strategies.
